# Artificial intelligence in colonoscopy: A review on the current status

**DOI:** 10.1002/deo2.109

**Published:** 2022-03-23

**Authors:** Solveig Linnea Veen Larsen, Yuichi Mori

**Affiliations:** ^1^ Clinical Effectiveness Research Group, Institute of Health and Society, University of Oslo Oslo Norway; ^2^ Department of Transplantation Medicine, Oslo University Hospital University of Oslo Oslo Norway; ^3^ Digestive Disease Center, Showa University Northern Yokohama Hospital Kanagawa Japan

**Keywords:** colorectal cancer, colorectal polyps

## Abstract

Artificial intelligence has become an increasingly hot topic in the last several years, and it has also gained its way into the medical field. In recent years, the application of artificial intelligence in the gastroenterology field has been of increasing interest, particularly in the colonoscopy setting. Novel technologies such as deep neural networks have enabled real‐time computer‐aided polyp detection and diagnosis during colonoscopy. This might lead to increased performance of endoscopists as well as potentially reducing the costs of unnecessary polypectomies of hyperplastic polyps. Newly published prospective trials studying computer‐aided detection showed that the assistance of artificial intelligence significantly increased the detection of polyps and non‐advanced adenomas approximately by 10%, while three tandem randomized control trials proved that the adenoma miss rate was significantly reduced (e.g., 13.8% vs. 36.7% in one Japanese multicenter trial). Promising results have also been shown in prospective single‐arm trials on computer‐aided polyp diagnosis, but the evidence is insufficient to reach a conclusion.

## INTRODUCTION

Colorectal cancer (CRC) is the third most diagnosed cancer in the world, with the second‐highest cancer mortality rate.^1^ Most CRCs develop sporadically from colorectal adenomatous polyps, and colonoscopy is established as the gold standard for the detection of these lesions. Colonoscopy is also considered the only screening technique that provides both a diagnostic and therapeutic effect, and it has been shown that endoscopic polypectomy of these pre‐cancerous lesions reduces the incidence and mortality of CRC. However, the quality of the colonoscopy is a determining factor in the diagnosis, and the adenoma detection rate (ADR) may vary greatly depending on the operator.^2^, ^3^

Artificial intelligence (AI) is being increasingly adopted in medical diagnostics, and in recent years, it has also paved its way into the gastroenterology field, particularly in the colonoscopy setting. Novel techniques such as deep neural networks have enabled the use of computer‐aided polyp detection (CADe) and diagnosis (CADx), potentially leading to increased quality of endoscopy and reduction of unnecessary polypectomies. These concepts have been studied in prospective trials in the last couple of years, generally concluding with increased detection of polyps and non‐advanced adenomas, as well as a significant reduction in the adenoma miss rate (AMR). However, improvement of the advanced adenoma detection rate (A‐ADR) has yet to be proven. The use of CADx has also been studied in prospective single‐arm trials, showing promising results, but still, the evidence is quite scarce. Nevertheless, encouraging results so far will with no doubt lead to increased exploration of this area, potentially paving the way for a completely new strategy in the colonoscopy setting.

The aim of this review is to assess the current status of AI in the colonoscopy setting and to review the results of relevant prospective studies on these topics.

### Quality indicators for colonoscopy

As colonoscopy is a procedure performed by humans, there is a natural variation in the quality of the colonoscopy. According to the European Society of Gastrointestinal Endoscopy guidelines from 2017, there are seven key performance measures relevant for the quality of lower gastrointestinal endoscopy.^4^ These include the rate of adequate bowel preparation, cecal intubation rate, ADR, and appropriate polypectomy technique, among others. They also include minor performance measures such as withdrawal time, polyp detection rate (PDR), and polyp retrieval rate.

Adenomas are major precursor lesions for CRC, and ADR is, therefore, a key quality indicator for colonoscopy, particularly in the screening setting. The ADR is a measure of the skills of individual endoscopists to detect adenomas during a colonoscopy procedure, and there is a great variation between endoscopists (e.g., 7,4% vs. 52,5% in one US study).^5^ ADR has been shown to be inversely related to the risk of interval CRC and mortality,^4^ which is also proven in the same US study, which reported a 3% decrease in the risk of interval CRC for every 1% increase in ADR.^5^ A Polish study demonstrated that an ADR above 24.6% was a prerequisite for reducing the risk of interval CRC, which is in line with the current recommendation targeting an ADR of at least 25% for a mixed male/female population.^4^, ^6^

Withdrawal time is also a widely used quality indicator, particularly for screening colonoscopy, and it is closely related to ADR. The withdrawal time is used to identify pathology during withdrawal from the cecum to the anal canal. A mean withdrawal time of at least 6 min is associated with a higher ADR and subsequently reduced risk of interval CRC. Hence, withdrawal time is also inversely related to the interval CRC risk.^4^ A large population‐based analysis studying the relationship between these two quality indicators actually proved that a –1‐min increase in withdrawal time led to a 3.6% absolute increase in the ADR.^7^ Even though not as high a yield as the ADR, the withdrawal time remains a useful quality indicator, particularly in settings where the observed ADR is less than the minimum recommendation of 25%.^4^

Although many more measures are important factors for the quality of colonoscopy, ADR and withdrawal time are particularly interesting, as they can potentially be improved with the assistance of AI. This suggests that the use of AI in the colonoscopy setting might lead to increased performance of endoscopists, thereby providing a more standardized and equal offer for all patients, and then again potentially aid in the reduction of interval CRC and mortality in the long run.

### CADe and CADx

Novel deep learning techniques have made it possible for endoscope manufacturers to develop specific AI tools in the colonoscopy setting, enabling real‐time polyp detection and diagnosis. The interest in applying AI to colonoscopy has also been strong among individual gastroenterologists, particularly regarding CADe.^8^ While CADe aims to decrease the rate of missed polyps during colonoscopy and ultimately increase the performance of the endoscopists, CADx has the property of real‐time interpretation of the polyp optical diagnosis, potentially being able to reduce the rate of unnecessary polypectomies of non‐neoplastic lesions.^9^ Regarding the cost‐effectiveness of AI, CADe, and CADx might create different scenarios, particularly on a short‐term basis. Implementation of CADe might further increase the short‐term cost as more polyps are detected, which again leads to increased rates of polypectomies, pathological examinations, and ultimately surveillance colonoscopies. CADx, on the other hand, might in fact decrease colonoscopy‐related costs, as it might lead to the reduction of unnecessary polypectomies, thereby stopping the subsequent cost requiring a chain of events. In the long term, CADe might also prove to be cost‐effective, as the increased detection rate can lead to a reduced incidence of interval CRC. Additional long‐term follow‐up data are required to decide whether the use of AI assistance is more cost‐effective than standard colonoscopy. Regarding the clinical effectiveness of AI in the colonoscopy setting, an answer might be in the near future, and several trials have already been published on this matter.

### CADe and computer‐aided quality improvement

Going back roughly two years in time, CADe had only been studied in a retrospective manner, using videos and images as test sets.^10^, ^11^, ^12^ As the results were increasingly positive, testing in real‐time became increasingly attractive and was also a necessary next step to clearly understand the effect of automated polyp detection in colonoscopy.

In the last couple of years, automated polyp detection has been studied in several prospective trials looking primarily at AMR and ADR. Three relatively new tandem randomized controlled trials (RCTs) published in 2020 and 2021 demonstrated that AMR was significantly decreased in the CADe group compared to the standard colonoscopy group^13^, ^14^, ^15^ (e.g., 13,8% vs. 36,7% in a Japanese multicenter trial and 20.12% vs. 31.25% in a US multicenter trial). Additionally, an increasing number of prospective trials studying the effect of AI on ADR have been published, mainly from China^16^, ^17^, ^18^, ^19^, ^20^, ^21^, ^22^, ^23^ but also from Italy conducted in a multi‐center fashion.^24^ The studies were published between 2019 and 2021, and the colonoscopy indications included screening, symptomatic, and surveillance patients. All the studies were RCTs conducted in a single‐center fashion, except for the Italian study, which included three centers. The number of included patients ranged approximately from 150 to 1000. Six of the studies aimed to assess the efficacy of CADe systems on the detection rate of colorectal polyps and adenomas^17^, ^19^, ^20^, ^22^, ^23^, ^24^, while one study constructed a real‐time quality improvement system to monitor the withdrawal time with the primary endpoint of ADR.^16^ Additionally, Su et al. conducted a study for both quality control and polyp identification.^18^ While the quality control studies of the withdrawal time did not directly aim at polyp identification, they evaluated the colonoscopy quality with the assistance of AI. Recently, a research group from Wuhan University in China published a four‐group parallel study comparing the ADR of CADe and computer‐aided quality improvement (CAQ) interaction.^21^ Participants were randomly assigned to either a control group, CADe group, CAQ group, or CADe plus CAQ (COMBO) group. They concluded that CAQ significantly improved the efficacy of CADe, with an ADR of 30.6% compared to 21.27% with CADe only. No significant difference was found between the CAQ and COMBO groups. The other RCTs conducted on the use of CADe mainly concluded that the assistance of AI in colonoscopy significantly increased the detection rate of polyps and non‐advanced adenomas approximately by 10%.^25^, ^26^ However, the detection rate of advanced adenomas remained unchanged. Nevertheless, these results were the most encouraging and certainly, pave the way for further exploration in this area. The next relevant step will be to conduct larger‐scale RCTs preferably with long‐term follow‐up to fully understand the effect of AI on the quality of colonoscopy.

### Computer‐aided polyp diagnosis

Automated polyp diagnosis has also been studied in several retrospective trials, as well as a few prospective single‐arm trials in recent years. For the retrospective studies, several methods for the application of CADx were studied, including magnifying narrow‐band imaging (NBI), magnifying chromoendoscopy, endocytoscopy, confocal endomicroscopy, laser‐induced fluorescence spectroscopy, autofluorescence endoscopy, and white light endoscopy. Among these methods, magnifying NBI has been most actively investigated and was first reported by Tischendorf et al.^27^ and Gross et al.^28^ back in 2009 and 2011. Their studies reported diagnostic accuracy of 85.3% and 93.1%, respectively, which proved that CADx of colon polyps could achieve high diagnostic performance, but according to Tischendorf et al., diagnosis by observers was still superior, and further research was required to clarify whether the CADx system could be improved.

In more recent years, a limited number of prospective single‐arm studies have been published on automated polyp diagnosis, mainly studying methods such as autofluorescence and NBI.^29^, ^30^, ^31^, ^32^, ^33^, ^34^ The studies were published between 2013 and 2018; hence, the development of this field has been much slower than automatic polyp detection.

Aihara et al.^29^ and Horiuchi et al.^30^ studied the use of real‐time CADx by using autofluorescence color analysis and looking at the lesions green/red (G/R) ratio. The first study included a total of 32 patients in which evaluation was performed on 102 colorectal lesions. A cutoff value of 1.01 G/R ratio was applied for discriminating between neoplastic lesions and non‐neoplastic lesions and had a sensitivity, specificity, positive predictive value, and negative predictive value (NPV) of 94.2%, 88.9%, 95.6%, and 85.2%, respectively.^29^ The second study included 95 patients with 258 diminutive rectosigmoid polyps and 171 diminutive non‐rectosigmoid polyps. This study demonstrated an accuracy of 91.5% for differentiating diminutive rectosigmoid neoplastic polyps.^30^

Another two studies aimed to evaluate the diagnostic performance of a novel automated polyp diagnosis system based on laser‐induced autofluorescence and fluorescence spectroscopy.^32^, ^34^ They evaluated the performance of WavSTAT and WavSTAT4 for their ability to predict polyp histology, including a total of 87 patients with 207 small colorectal lesions^32^ and 27 patients with 137 diminutive colorectal polyps.^34^ The first study showed an accuracy of 74.4%, which was insufficient for the differentiation of small colorectal lesions.^32^ The second study, however, had an overall accuracy of 84.7%, which proved to be sufficient to allow distal colorectal lesions to be left in place and almost reached the threshold for a “resect and discard” method.^34^

The most recent study on automatic polyp diagnosis was published in 2018 and evaluated a real‐time CADx system with ultramagnifying colonoscopes providing microvascular and cellular visualization of colorectal polyps with the application of NBI and methylene blue staining modes, respectively.^33^ A total of 466 diminutive polyps from 325 patients were assessed by CADx, proving an NPV of 96.4% (best‐case scenario) and 93.7% (worst‐case scenario) for diminutive rectosigmoid adenomas after stained mode application and 96.5% (best‐case scenario) and 95.5% (worst‐case scenario) with NBI. These results proved that real‐time CADx can reach the threshold required for a “diagnose‐and‐leave” strategy (NPV ≥ 90%) for diminutive, non‐neoplastic rectosigmoid lesions.^33^

The outcomes of these studies are mostly encouraging and could pave the way for new strategies, such as the resect and discard strategy and the diagnose and leave strategy. This may provide great benefits in terms of cost‐effectiveness, workload, time, and patient burden. Nevertheless, the evidence of CADx is still scarce and has yet to be evaluated in larger‐scale randomization studies to further clarify its benefits vs. risks.

## CONCLUSION

Randomized studies on computer‐aided detection and computer‐aided quality improvement have proven to increase the detection rate of polyps and non‐advanced adenomas but remain to show an effect on the detection rate of advanced adenomas. Computer‐aided polyp diagnosis has shown promising results that might encourage the use of new strategies, such as the resect and discard and diagnose and leave strategy. Nevertheless, evidence is still scarce on this topic, and further studies should be performed before reaching a conclusion, preferably larger‐scale randomized trials.

## CONFLICT OF INTEREST

Yuichi Mori has received a Consultant fee from Olympus Corp. and Cybernet Corp. Yuichi Mori is Associate Editor of DEN Open.

## FUNDING INFORMATION

None.
